# Late Onset Neurodegeneration with Brain-Iron Accumulation Presenting as Parkinsonism

**DOI:** 10.1155/2012/387095

**Published:** 2012-12-18

**Authors:** Robert Fekete

**Affiliations:** Department of Neurology, New York Medical College, Munger Pavilion, 4th Floor, 40 Sunshine Cottage Road, Valhalla, NY 10595, USA

## Abstract

Neurodegeneration with brain-iron accumulation (NBIA) encompasses a family of neurodegenerative disorders connected by evidence of abnormal brain iron deposition. Advances in imaging and genetic testing expanded the clinical spectrum of these disorders. Here, a case of parkinsonism and dystonia with orofacial stereotypies is presented. While the patient was initially diagnosed with Parkinson's disease and placed on levodopa therapy, dopamine transporter imaging via (123)I-FP-CIT SPECT (DaTSCAN) was normal. MRI brain showed “eye of the tiger” sign on T2 weighted imaging. NBIA should be considered in the differential diagnosis of atypical parkinsonism.

## 1. Introduction

Neurodegeneration with brain-iron accumulation (NBIA), formerly called Hallervorden-Spatz syndrome, encompasses a family of neurodegenerative disorders connected by evidence of abnormal brain-iron deposition.

NBIA was first identified anatomically via rust colored iron deposits in the globus pallidus and substantia nigra and typically consists of a movement disorder including dystonia and chorea, possible spasticity, and cognitive disturbance [1]. Advances in imaging and genetic testing have allowed ante mortem diagnosis and expanded the known phenotypic spectrum of NBIA. Among these has been the phenotype of late onset parkinsonism [2–4]. Here, a case of NBIA initially diagnosed as Parkinson's disease is described. NBIA should be considered in the differential diagnosis of atypical parkinsonism.

## 2. Case Presentation

The patient is a 75-year-old right handed male who presented with two year history of asymmetric rest tremor, worse on the right, slowness of movement, and gait instability. He was diagnosed with Parkinson's disease a year ago and started on levodopa therapy without significant benefit. At time of the initial visit, he required a cane to ambulate.

Past medical history is significant for hypertension, diabetes mellitus, and ten years of iron deficiency anemia and constipation. Given worsening of constipation on oral iron supplementation, he was no longer on iron replacement therapy.

He denied a family history of movement disorders. There was no orthostatic hypotension or other evidence of dysautonomia even on levodopa except for constipation.

On examination, there was no evidence of aphasia. There was mild impairment of remote recall. There was dysarthria, likely due to continuous orofacial dyskinesia. Pursuit was mildly saccadic and there was mild impairment of horizontal and vertical saccade velocity and initiation. There was an in intermittent right thumb flexion/extension rest tremor at about 8 Hertz frequency. Left thumb flexion/extension rest tremor was seen when the patient was handwriting or performing repetitive movements with the right hand. There were occasional finger tapping stereotypies of both hands. There was right worse than left upper and lower extremity bradykinesia and rigidity. There were no upper motor neuron signs. There was a slight kinetic tremor on finger-to-nose testing as well as mild flexion/extension postural tremor without latency at the metacarpophalangeal joints bilaterally. Gait was profoundly bradykinetic with start hesitation and freezing during turns. Freezing improved with use of visual cues.

Dopamine transporter imaging via single photon emission computerized tomography (SPECT) with ^123^I-N-fluoropropyl-2b-carbomethoxy-3b-(4-iodophenyl) nortropane (^123^I-FP-CIT)-DaTSCAN was normal. Levodopa was stopped given this result and perceived lack of efficacy. Stopping levodopa unmasked periodic bouts of dystonic cramping of neck and hands. In retrospect, the patient may have had a levodopa responsive dystonic phenotype. The orofacial dyskinesia and bilateral upper extremity stereotypies continued even off levodopa.

The patient had iron deficiency anemia (serum iron low at 32 mcg/dL, hemoglobin 10.2 g/dL, and hematocrit 31.5%) with negative fecal occult blood test and low ferritin (14 mcg/dL). MRI abdomen and pelvis did not find any masses. There was no evidence of abnormal signal in the liver. Serum paraneoplastic antibody panel was negative.

**Figure 1:**
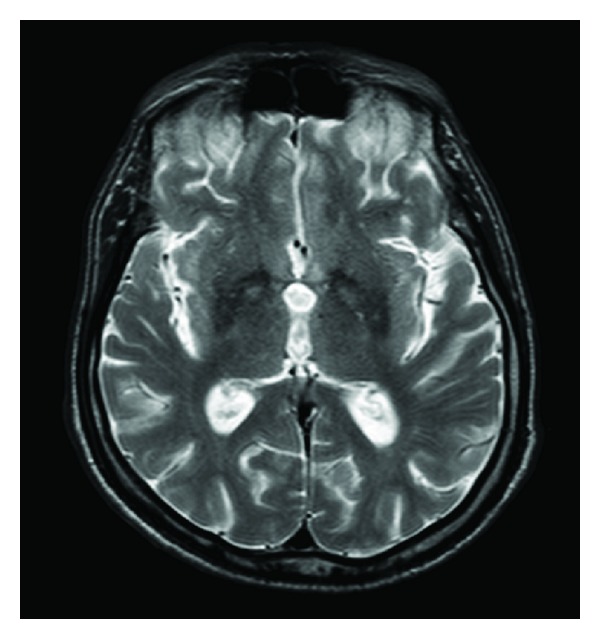
Axial T2 weighted MRI image showing “eye of the tiger” sign with bilateral hypointense rim of globus pallidus and area of central hyperintensity, in addition to hypointense posterolateral putamen.

**Figure 2:**
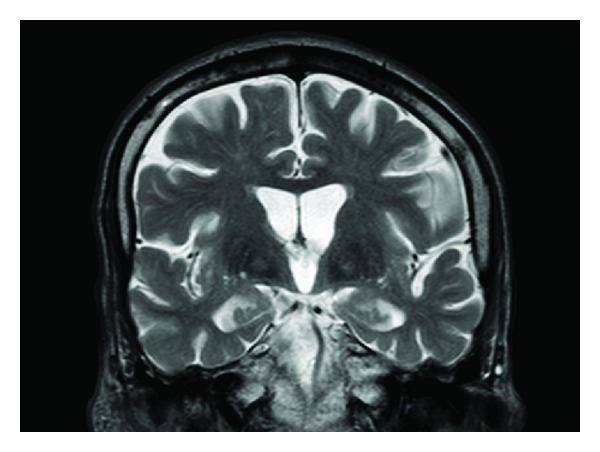
Coronal T2 weighted MRI image showing bilateral “eye of the tiger” sign in globus pallidus as well as bilateral putaminal hypointensity. While there are some areas putaminal T2 hyperintensity, they are not clearly organized into a prominent lateral hyperintense rim and adjacent discrete hypointensity as in MSA.

MRI brain revealed “eye of the tiger” sign on T2 weighted axial and coronal images (Figures 1 and 2). T2 hypointensity extended from the globus pallidus in to the putamen bilaterally. In contrast to this case, NBIA cases due to PANK2 mutation have T2 abnormalities confined to the globus pallidus [5]. More diffuse T2 involvement is seen with other types of NBIA, for example putamen and dentate nuclei in neuroferritinopathy. In addition, acanthocytosis, typically associated with PANK2 mutation, was not observed on blood smear. Given the low ferritin, putaminal involvement, and orofacial stereotypies, FTL gene sequencing for suspected neuroferritinopathy was performed. FTL gene sequencing was normal, which fit the absence of any autosomal dominant inheritance pattern typically seen in this disorder. Hence, he was diagnosed with NBIA, unknown type, and restarted on levodopa to control dystonia. Intravenous iron was successfully used to treat his anemia.

## 3. Discussion

The patient's imaging, laboratory, and clinical presentation was compared with known types of NBIA. NBIA due to neuroferritinopathy was considered as discussed above but ultimately ruled out given normal FTL genetic test.

NBIA Type 1, panthotenate kinase associated neurodegeneration (PKAN), formerly called Hallervorden-Spatz disease, was discovered to be caused by the PANK2 gene. It is typically a young onset recessively inherited movement disorder with cognitive disturbance, but late onset parkinsonism with tremor was described by a PANK2 mutation positive family by Thomas et al. [4] Putaminal T2 hypointensity and lack of acanthocytes argue against a diagnosis of PKAN in this case.

PLA2G6 mutation (NBIA Type 2) causes infantile neuroaxonal degeneration (INAD), but recently, cases of adult onset PLA2G6 associated neurodegeneration (PLAN) have been described [6]. Tan and colleagues discuss a patient with dystonia-parkinsonism with onset at age 69 who had a PLA2G6 mutation but did not have evidence of iron deposition on CT scan [7]. As patients with this mutation lack the “eye of the tiger” sign on MRI and instead may present with either normal MRI or T2 hypointensity in the globus pallidus without hyperintensity, PLAN was not considered in this case [8].

The patient had normal serum ceruloplasmin (26 mg/dL), normal serum copper (85 mcg/dL), and low ferritin which argue against aceruloplasminemia. In that disorder, serum ceruloplasmin is usually undetectable, serum copper and iron are low, and ferritin is elevated [8]. There was no abnormal deposition of iron in the liver on MRI of the abdomen.

While an abnormality similar to “eye of the tiger” has been reported in multiple system atrophy (MSA) [9], this patient did not experience the profound dysautonomia typical of MSA. In addition, the reported cases of MSA have a less hypointense rim of the globus pallidus due to absence of iron deposition. Dopamine transporter imaging would have been expected to be abnormal in MSA but was normal in this case. A clearly defined T2 hyperintense lateral rim of the putamen with adjacent hypointensity is typical for MSA, but was not seen in this case.

The patient may have NBIA due to a yet undescribed mutation. Further genetic advances will allow for a better understanding of late onset dystonia-parkinsonism due to NBIA.
